# Depth Estimation of Submerged Aquatic Vegetation in Clear Water Streams Using Low-Altitude Optical Remote Sensing

**DOI:** 10.3390/s151025287

**Published:** 2015-09-30

**Authors:** Fleur Visser, Kerst Buis, Veerle Verschoren, Patrick Meire

**Affiliations:** 1 Institute of Science and the Environment, University of Worcester, Henwick Grove, Worcester WR2 6AJ, UK; 2 Department of Biology, Ecosystem Management Research Group, University of Antwerp, Universiteitsplein 1C, Wilrijk B–2610, Belgium; E-Mails: kerst.buis@uantwerpen.be (K.B.); veerle.verschoren@uantwerpen.be (V.V.); patrick.meire@uantwerpen.be (P.M.)

**Keywords:** submerged aquatic vegetation, macrophytes, fluvial, remote sensing, submergence depth, bathymetry, OBRA

## Abstract

UAVs and other low-altitude remote sensing platforms are proving very useful tools for remote sensing of river systems. Currently consumer grade cameras are still the most commonly used sensors for this purpose. In particular, progress is being made to obtain river bathymetry from the optical image data collected with such cameras, using the strong attenuation of light in water. No studies have yet applied this method to map submergence depth of aquatic vegetation, which has rather different reflectance characteristics from river bed substrate. This study therefore looked at the possibilities to use the optical image data to map submerged aquatic vegetation (SAV) depth in shallow clear water streams. We first applied the Optimal Band Ratio Analysis method (OBRA) of Legleiter *et al.* (2009) to a dataset of spectral signatures from three macrophyte species in a clear water stream. The results showed that for each species the ratio of certain wavelengths were strongly associated with depth. A combined assessment of all species resulted in equally strong associations, indicating that the effect of spectral variation in vegetation is subsidiary to spectral variation due to depth changes. Strongest associations (R^2^-values ranging from 0.67 to 0.90 for different species) were found for combinations including one band in the near infrared (NIR) region between 825 and 925 nm and one band in the visible light region. Currently data of both high spatial and spectral resolution is not commonly available to apply the OBRA results directly to image data for SAV depth mapping. Instead a novel, low-cost data acquisition method was used to obtain six-band high spatial resolution image composites using a NIR sensitive DSLR camera. A field dataset of SAV submergence depths was used to develop regression models for the mapping of submergence depth from image pixel values. Band (combinations) providing the best performing models (R^2^-values up to 0.77) corresponded with the OBRA findings. A 10% error was achieved under sub-optimal data collection conditions, which indicates that the method could be suitable for many SAV mapping applications.

## 1. Introduction

Increasingly Unmanned Aerial Vehicles (UAVs) and other low-altitude remote sensing platforms such as kites and telescopic poles are used to map the spatial distribution of fluvial properties for management, monitoring, and modelling of river systems. These low-altitude remote sensing approaches overcome issues of spatial and temporal coverage, which particularly affect application of conventional air and spaceborne remote sensing to smaller river systems. An important limitation of such platforms is their small payload, which means that the most commonly used sensors are consumer grade photo cameras with a low spectral resolution and range. This warrants further research to find out how this type of sensors can be used to map the spatial distribution of fluvial properties.

The presence of submerged aquatic vegetation (SAV) can play a dominant role in influencing flow conditions in lowland river systems. It affects stream flow heterogeneity, hydraulic resistance, and sediment retention [1,2,3] and is therefore of importance for flood management. Furthermore the patchiness of macrophytes creates a heterogeneous environment determining stream ecosystem functioning [4]. In some cases it can become invasive by forming extensive canopies, which may affect light penetration, navigation, recreation, and fisheries [5]. Submergence depth and extent of SAV cover are fluvial properties of interest for the development of models that can give insight in these impacts of SAV abundance. Where remote sensing techniques can achieve great detail and accuracy for 3D mapping of terrestrial vegetation [6], obtaining bathymetry of SAV at a similar scale (<1 m) is much more difficult in aquatic environments. This is largely due to the interaction between water and electromagnetic radiation at the wavelengths most suitable for this kind of analysis. A number of authors have published overviews of bathymetric mapping methods and their application to rivers [7,8,9]. The two most suitable approaches described by these authors for obtaining high resolution information of smaller, shallow river systems, are those using spectral-depth relationships and those using photogrammetric methods, including structure from motion (SfM) approaches [10]. The spectral-depth relationship approach is currently most commonly used and has been performed using multispectral, RGB (true colour), and black and white imagery obtained with standard photo cameras (e.g., [7,11,12,13]).

The spectral-depth relationship approach makes use of the exponential relationship between image-derived reflectance and water depth through regression analysis, as first suggested in [14] and since then commonly applied in marine and fluvial environments. It is based on the principle that bottom reflectance measured above the water surface will be reduced due to attenuation of light in water. The strength of the reflectance signal is therefore related to depth of the bottom below the water surface and can be used to determine bathymetry [8,11,15,16]. The optimal wavelengths for the band ratio-based algorithms used in most of these studies, however, have been determined for streams without (significant) SAV growth. A problem noted by several authors is the effect of variation in bottom substrate and presence of SAV cover on depth estimates [13,17]. Similar issues were found for seagrass in shallow coastal environments by [18]. In contrast to non-photosynthetic bottom material, vegetation generally has high NIR reflectance like terrestrial vegetation. Due to the strong attenuation of light in these wavelengths by water, the optimal wavelength bands for depth estimates of SAV are likely to be different compared to bare substrate.

The aim of the project presented in this paper is to investigate the possibility of creating maps of SAV depth distribution in shallow clear water streams from images obtained with a consumer grade digital camera using spectral-depth relationships. This is done in two stages:

Firstly we determine how vegetation spectral signatures, obtained by means of field spectroscopy, relate to water depth using the Optimal Band Ratio Analysis (OBRA) method, as developed by [15]. With this method it is possible to determine the most suitable wavelength band combinations for depth retrieval from high spectral resolution reflectance data.

Secondly we apply the spectrally based depth retrieval approach to multi-spectral image composites obtained with a NIR sensitive consumer grade camera to map SAV depth and extent for 6–8 m long reaches of a Belgian clear water stream. The OBRA method was developed for use with data of hyperspectral resolution. Although hyperspectral data of decimeter resolution can be obtained, such spatial resolution is not sufficient for detailed mapping of SAV depth and extent small rivers, while the cost of obtaining such imagery is still very high and logistics are difficult. This means that wavelength combinations identified with the OBRA method cannot be used directly to obtain spatial information of SAV depth distribution. Instead two to six-band multi-spectral image composites were created using a NIR sensitive camera elevated from a telescopic mast. The resulting SAV maps are discussed in the light of findings from the OBRA study.

## 2. Methods

### 2.1. Study Site

Most of the data for the OBRA analysis were obtained from the River Wylye where it flows through the Langford Trust nature reserve in Wiltshire, UK, during two fieldwork periods in August/September 2009 and 2010. Some additional data for this part of the project came from a distributary of the River Frome near Wool in Dorset. Both sites are typical English chalk streams and physically very similar, with a stream width of around 5 m, a maximum water depth at time of sampling of around 0.5 m and a mean discharge of approximately 0.3 m^3^·s^−1^. The water in the steams has exceptionally low turbidity (<10 mg/L during the fieldwork period) and contains an abundant macrophyte cover, with Water Crowfoot (*Ranunculus fluitans*, Lam), Fennel Pondweed (*Potamogeton pectinatus*, L.), and Spiked Water Milfoil (*Myriophyllum spicatum*, L.) as some of the most common species at the sites used.

The data for the vegetation mapping were collected in May 2012 from three locations along the Desselse Nete close to its confluence with the Zwarte Nete near the village Retie in Belgium. The stream has generally low suspended solid and organic matter concentrations (<50 mg/L). The stream width is 6.2 m, with an average water depth of 0.5–0.6 m and mean discharge of 0.35–0.6 m^3^·s^−1^. The vegetation on one sample site along this stream consists of mainly dense patches of Water Crowfoot (*Ranunculus aquatilis*, L) and Blunt-fruited Water Starwort (*Callitriche obtusangula*, *Le Gall*). The plants on two other sample sites are more open and consist of Broad-Leaved Pondweed (*Potamogeton natans*, L.) and European Bur-reed (*Sparganium emersum*, L.).

### 2.2. OBRA: Introduction

Legleiter *et al*. [15,19] provide a clear explanation of the theoretical background behind spectrally based depth retrieval. They conclude that the commonly used deep-water correction or Lyzenga algorithm [14] is unsuitable for shallow river conditions and suggest a modified approach. The Lyzenga algorithm requires generally unavailable knowledge of the amount of radiance from optically deep water. Instead [15] assume that the amount of radiance from constituents of the water column, from surface-reflected radiance and from radiance derived from the atmosphere between sensor and fluvial target, are insignificant compared to the amount of radiance coming from the river bottom in shallow and relatively clear streams. These radiance sources also further diminish as stream depth decreases. Various authors have taken the approach of using the ratio of at sensor radiances *L_T_(λ)* in two wavelength bands, referred to as *X*, so that the ratio of the bottom reflectance in these two bands is the same for all the bottom types, but still dependent on depth [20,21]. For shallow river conditions according to the assumptions by [15] this will correspond with the ratio of bottom reflected radiance *L_b_(λ)* in the two bands:

(1) $$X=\text{ln}\left[\frac{L_{\text{T}1}}{L_{\text{T}2}}\right]≅\text{ln}\left[\frac{L_{\text{b}1}}{L_{\text{b}2}}\right]$$

This equates with a simplified version of the Lyzenga algorithm, which forms a dimensionally homogeneous, linear relationship between the (image) data-derived variable X and water depth *d*:

(2) $$X≅\left(K_{2}-K_{1}\right)d+\text{ln}\left[\frac{\left(R_{b1}-R_{c1}\right)}{\left(R_{b2}-R_{c2}\right)}\right]+A]$$

With:

(3) $$A=\text{ln}\left[\frac{{\text{E}}_{\text{d}1}{\text{C}}_{1}{\text{T}}_{1}}{{\text{E}}_{\text{d}2}{\text{C}}_{2}{\text{T}}_{2}}\right]$$

*K(λ)* in Equation (2) are the effective attenuation coefficients; *R_b_(λ)* bottom reflectance of the river and *R_c_(λ)* volume reflectance of the water column. The slope term (*K*_2_ − *K*_1_) represents the difference in effective attenuation between two bands. *X* increases with depth if *K*_2_ is bigger than *K*_1_. The intercept incorporates the bottom contrast between the streambed and the water column, as well as constant *A* (Equation (3)), which is determined by downwelling solar irradiance *E_d_(λ)*, transmission across air–water interface *C(λ)* and transmittance of the atmosphere *T(λ)*. Apart from water depth *d* the variables in this equation are all assumed constant throughout a river reach (*i.e.*, Both *K(λ)* and *R_c_(λ)*, which are determined by the inherent optical properties of the water column; *R_b_(λ)* depends on substrate composition, but *R_b_*_1_*/R_b_*_2_ is assumed constant across bottom types and the ratio will therefore not vary spatially). Consequently the equation can be used to estimate *d* on a pixel by pixel basis, based on the remotely sensed variable *X*. Bottom reflected radiances *L_b_*_1_ and *L_b_*_2_ are directly derived from image data by extracting pixel digital numbers (DN) for two bands, as the difference and ratio based calculations of the equation slope and intercept make precise knowledge of absolute radiance values unnecessary.

Based on the above theoretical development [15] propose the OBRA method to determine the optimal band ratio that should be used to map bathymetry from passive optical image data. OBRA calculates *X* for each pair of bands (*λ*_1_, *λ*_2_) and determines its association with *d*. The resulting coefficient of determination R^2^-values are plotted in an *n* × *n* matrix (where *n* equals the number of measured wavelength bands). Only the bottom half of the matrix is retained as the results are symmetrical on either side of the diagonal. The spread of R^2^-values in the matrix indicates which bands yield the strongest relationships with depth and how unique these are.

Legleiter *et al.* [15] were the first to investigate the effect of substrate on the selection of optimal wavelengths. For river beds with gravel and periphyton substrates they found that the ratio of reflectances at 586 and 614 nm were strongly related to depth (R^2^ = 0.945). However, the authors commented that the range of suitable wavelengths for depth estimates might be limited in the presence of more spectrally distinct substrates. None of their studied sites had an extensive vegetation cover.

### 2.3. OBRA: Data Collection

**Figure 1:**
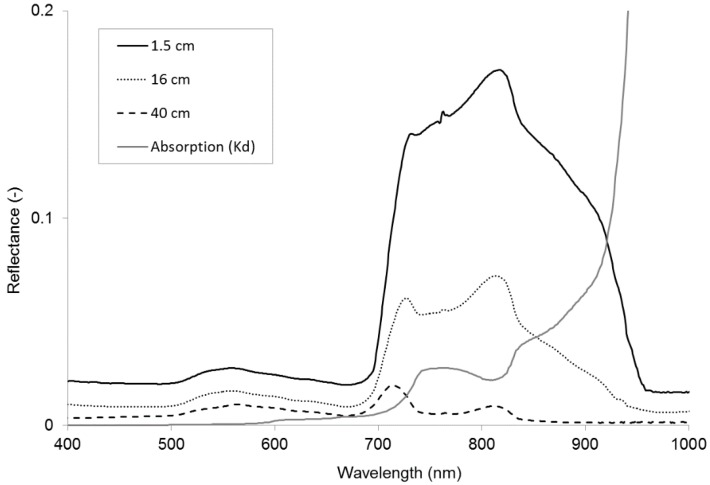
Reflectance spectra of Water Crowfoot at 1.5, 16, and 40 cm below the water surface and the absorption coefficients of water (cm^−1^) for wavelengths between 400 and 1000 nm.

Our input for OBRA consists of *in situ* point measurements of submergence depth values and reflectance spectra for three macrophyte species Water Crowfoot, Fennel Pondweed, and Spiked Water Milfoil, collected with a GER1500 hand-held field spectroradiometer. Figure 1 shows examples of reflectance data obtained for Water Crowfoot. Hyperspectral resolution reflectance spectra are shown for three different submergence depths (1.5–40 cm). The spectra clearly show how reflectance from the macrophyte cover decreases with depth at variable rates depending on wavelength. Due to limited access to the river and limited availability of specific vegetation species at different depths purposive sampling was applied to obtain submerged vegetation spectra. The GER1500 was held at nadir 50 cm above the water surface. The instrument has a 3° field of view so the area measured on the target has a 2.6–4.0 cm diameter (depending on submergence depth), which is assumed sufficient to obtain representative spectral information from the dense vegetation stands. Sampling was carried out on cloud-free days within 2 h of solar noon. Spectral averaging of 10–30 spectra per sample was performed to ensure optimal signal-to-noise ratio. A white reference Spectralon calibration panel of 99% reflectance was used every 5 to 10 samples to offset any change in the atmospheric condition and irradiance of the sun. Reflectance was calculated by dividing macrophyte radiance by radiance from the Spectralon surface.

Table 1 shows a summary of the sample numbers and depth ranges for each of the SAV species. Each sample has a spectral range of 350–1050 nm and a sampling interval of 1.5 nm. Due to the relatively shallow water depths in chalk streams not all NIR radiation is absorbed. Suspended load is mostly absent from the sampled streams, so no water property adjustments were made.

**Table 1:** Submergence depth range and number of spectral samples taken from different macrophyte species.

SAV Species	N	Depth Range (cm)
Fennel Pondweed	60	10–25
Water Crowfoot	37	2–40
Water Milfoil	66	3–50

### 2.4. Depth Mapping: Image Data Collection and Preprocessing

**Figure 2:**
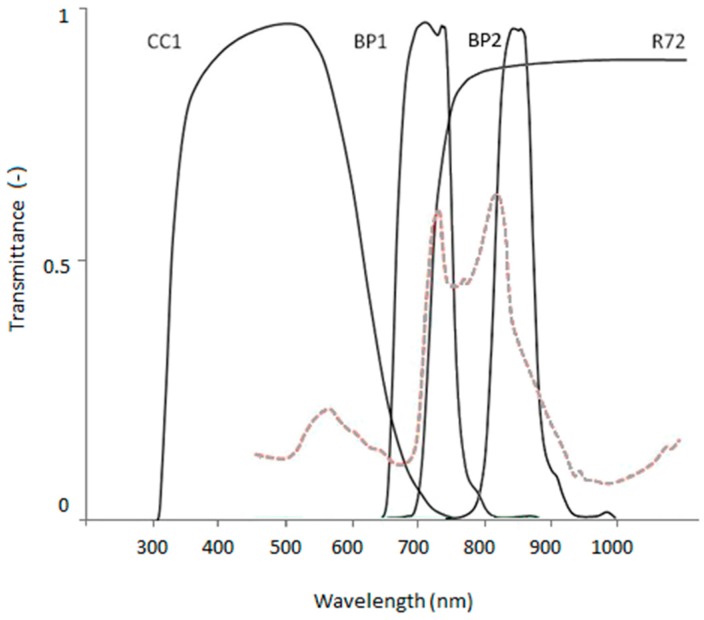
Transmission spectra of BP1 and BP2 bandpass filters and CC1 and R72 blocking filters based on manufacturers specifications (maxmax.com). Submerged macrophyte spectrum included with dashed red line for comparison.

To create vegetation submergence depth maps image data was collected from three locations along the Desselse Nete. One location was photographed on two different days; they will be referred to as site 1a and site 1b. The image data was obtained using a Fujifilm IS-Pro NIR sensitive DSLR camera with a Tamron AF Aspherical 28–80 mm f/3.5–5.6 lens attached to a pole and positioned approximately 4.5 m at nadir over the centre line of the river. The pole was secured on the river bank and held in position by guy ropes. Photos were taken with a radio controlled remote shutter in 3024 × 2016 pixels, 8-bit, GEOTIFF format. Although TIFF format is not thought to be most suitable [22] it was used in this case because of ease of use (format and file size). Multi-spectral image composites were created by taking a series of four photos from the same location, using different filters. Each filter transmits a specific part of the electromagnetic spectrum resulting in a distinct broad spectral band for the image composite. Red, Green, and Blue image bands were obtained by covering the camera lens with a NIR blocking filter and using the camera RGB channels. A visible light (VIS) blocking filter was used to obtain a single band covering most of the NIR spectrum (NIR(R72)) and two bandpass filters were used to obtain a narrow NIR wavelength band round 710 nm (NIR(BP1)) and 828 nm (NIR(BP2)). Figure 2 shows the filter transmission spectra and additional specifications are listed in Table 2. Relative ambient light conditions were estimated with an ATP DT-1309 Auto Ranging Light Meter.

**Table 2:** Specifications of lens filters used to obtain a six-band multi-spectral image.

Band Name	Filter Type	Transmission Characteristics	Estimated 50% Band Cuts
Blue	MaxMax X-Nite CC1 NIR blocking filter	centre: 483 nm; 50% transmission: 325 nm, 645 nm	400–500
Green			500–570
Red			570–645
NIR(R72)	Hoya R72 VIS blocking filter	>720 nm	720–1000
NIR(BP1)	MaxMax XNiteBPB band pass filter	5% low cut to 5% high cut: 650 nm to 787 nm 50% low cut to 50% high cut: 662 nm to 753 nm	662–753
NIR(BP2)	MaxMax XNiteBPG band pass filter	5% low cut to 5% high cut: 735 nm to 935 nm 50% low cut to 50% high cut: 795 nm to 860 nm	795–860

#### 2.4.1. Depth Mapping: Radiometric Correction

Before undertaking further image analysis radiometric and geometric pre-processing steps were applied to the image data. Firstly, removal of two types of radiometric anomalies seemed necessary, namely sunglint and a form of hotspotting or lens flaring. The latter is an anomaly caused by internal reflection of light within the camera, lens, and filter combination which occurred only in the NIR(BP2) band. The effect is known to occur quite frequently in the NIR wavelengths and some camera/lens/filter combinations are more prone to it than others. According to unverified sources and personal experience the occurrence is however also dependent on aperture settings and does not necessarily occur under all light conditions. The problem was not identified in the field so a flatfield image showing the same pattern, which would enable radiometric correction, was not produced on site. Attempts to recreate the effect at a later stage failed. Alternative correction methods as suggested in [23] were also not suitable due to the uniform scene content in our dataset. Instead the usefulness of applying an image based correction method to affected images was evaluated. This involved creating a continuous image correction mask, which represents the radiometric variation due to flaring. This was done by determining lowest image values along each of 90 concentric circles with increasing diameter and the same midpoint as the flare banding. Lowest image values for each circle were plotted and interpolated using a five-point moving average. The resulting spectral sinuosity was applied across the full image surface, resulting in a mask which was then subtracted from the original image.

For marine environments, models have been developed to remove sunglint from images of water surfaces. They mask glint based on sun-viewing geometry, using surface slope statistics. This approach is not suitable for high resolution data (<100 m) [24]. In fluvial environments surface topography is dependent on other factors than wind speed, while wind direction in the river channel will be unpredictable. Alternative methods using NIR/VIS differences are also not suitable for this situation as they assume little or no upwelling NIR radiance (e.g., from benthic vegetation) [24]. Instead sunglint is dealt with by excluding the highest DN values from the model calculations. Due to the relatively small depth/DN samples it was possible to manually check whether observations were removed from the samples for valid reasons (*i.e.*, being glint). Additional issues are caused by skyglint in the VIS images; however no adjustments were made for this effect.

#### 2.4.2. Depth Mapping: Geometric Correction

A commonly used form of geometric correction is lens barrel correction. No lens profile data could be found for the lens used in this project to perform this in image analysis software. Instead the distortion was assessed by photographing a regular grid and correcting the grid line curvature in Photoshop. This required a correction of less than 1% in both horizontal and vertical direction, which corresponds with the claims of Tamron (tamron-usa.com) that the aspherical lens type eliminates aberrations and distortion. No geometric correction was therefore applied before image co-registration.

All images for a site were co-registered using image-to-image tie points which consisted of four fixed ground control points that were included in all photos plus additional features identified in multiple photos. Second order polynomial transformations were applied which resulted in root mean squared errors (RMSE) ranging from 0.1 to 10 cells (≈ 0.2 to 24 mm). The larger errors are likely to be due to the fact that tie points in addition to the four ground control points were impossible to locate on the moving water surface and were difficult to find on the grassed banks. The final image layers were obtained by nearest neighbour resampling of the transformed data resulting in images with a resolution of 2.4 mm. Combinations of six different image layers were stacked into a single multi-band file. Parts of the scenes not covered by all image bands were cropped before further analysis.

### 2.5. Depth Mapping: Depth Data Collection

Immediately after collection of the multi-spectral image data, a set of submergence depth measurements was manually taken from the SAV within the camera field of view. This was done by outlining vegetation at a range of depths below the water surface with bamboo sticks. Sampled depths ranged from 0 to 60 cm below the water surface with intervals of 10 cm. The positions of the bamboo-sticks were photographed and the photos were co-registered with the multi-spectral image composite. In each photo circular polygons (app. 18.5 cm in diameter) were digitized at the depth measurement locations and used to extract spectral information for vegetation at that point from the multi-spectral composites. Image data samples extracted from within the polygons consist of, on average, 4000 pixels and are thought to represent a sufficiently large homogenous section of vegetation. From each of these spectral samples basic DN summary statistics (maximum, minimum, mean, and standard deviation) were calculated. The image and depth data collection was repeated for all four sites.

### 2.6. Depth Mapping: Analysis

The polygon DN statistics were used as equivalent to the total at sensor radiance to calculate variable *X* as in Equation (1). By doing this we followed the assumptions for clear shallow water that allow simplification of the total at sensor radiance and depth relationship, as suggested in [15]. Taking the ratio of two wavelength bands eliminates the effect of substrate variation as this would not have much influence on the ratio value, while the ratio would change with depth due to the difference in attenuation for different wavelengths. Because this study only looked at vegetation cover (*i.e.*, relatively little variation in spectral signatures amongst the cover compared to variation in signatures due to submergence depth), we also investigated the radiance-depth relationship using log-transformed DN values of individual bands. Although this will remove the advantages of substrate independence as well as possibly increased sensitivity to lighting conditions, the approach may be warranted, because the image composites used in this study were based on multiple photos. The implications of this will be considered in the discussion. The coefficients of determination for all individual bands and all band combinations will be presented in a 6 × 6 matrix representing each of the six image bands, similar to the OBRA plots used in the first phase of the project.

The linear models that best describe the water depth dependency of each band (combination) at each measurement site were then selected to create submergence depth maps from full images. The ability of these models to predict submergence depth from image DN values was validated in two ways: firstly, the model equations were used to estimate depth for a set of 15% of the sample points that were randomly selected from the data and had not been used in the model development. Secondly, the equations were used to estimate depth values from DN values of images from the other three measurement sites. The predicted depth values were compared to the modelled values, using a regression analysis. The R^2^, standard error, slope, and intercept of the resulting regression equations were used to assess the agreement between the observations. The final SAV bathymetric maps were post-processed using a 4 × 4 spatial mean filter to remove small scale noise (of unknown origin), before applying hill shading to enhance the Digital Elevation Model (DEM) display.

## 3. Results

### 3.1. OBRA Plots Based on Field Spectroscopy Data

**Figure 3:**
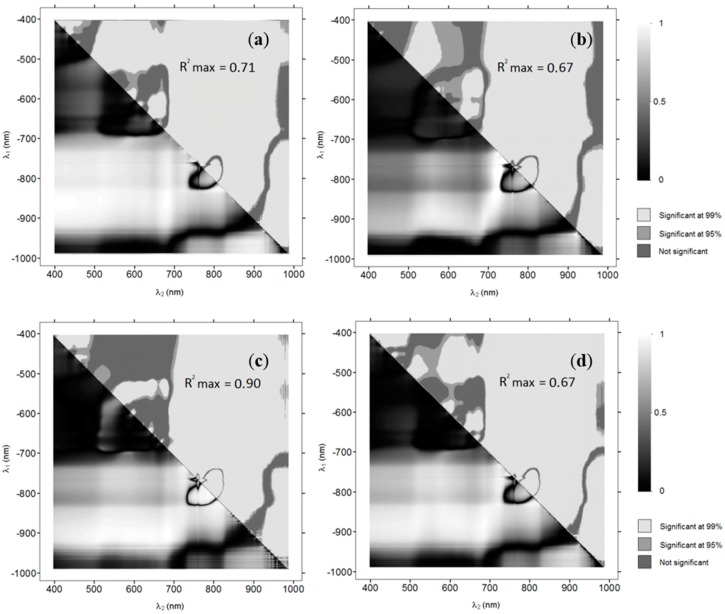
Optimal Band Ratio Analysis (OBRA) plots showing strength of image band ratio—depth associations for (**a**) Water Milfoil, (**b**) Pondweed, (**c**) Water Crowfoot, (**d**) all species. Bottom half R^2^-values; top half 99% significance level (light-grey) and 95% significance level (medium-grey).

Figure 3 shows the OBRA coefficient of determination values as images where row numbers represent the *X* numerator wavelengths (Equation (1)) and the column numbers, the denominator wavelengths. The shades in the bottom triangle of each image represent the OBRA R^2^(*λ*1, *λ*2) values. The shades in the top half indicate for each band combination the significance of the results with significance level of 95% in medium-grey and of 99% in light-grey. The top half of each image also contains the maximum R^2^-values (‘R^2^ max’) obtained for each species.

OBRA plots show rather similar patterns for each of the three species individually and for all species combined. All species combined, Water Crowfoot and Milfoil show the highest coefficient of determination values for band combinations with one band in the NIR region between 825 and 925 nm and one band in the VIS region (400 to 700 nm). The strong association of depth with band combinations including one band in the NIR region is to be expected as, in these wavelengths, water absorption strongly increases. It is further notable that the ranges of most suitable wavelength combinations vary slightly for the different vegetation types. The associations for Pondweed were less strong than for the other species. Association patterns throughout the NIR follow the water absorption curve (as shown in Figure 1), with a clear decrease in the coefficients of determination when both wavelengths fall between 765 and 810 nm corresponding with a slight reduction in water absorption, as well as an O_2_ absorption feature at 761 nm. Associations are not very strong in the VIS wavelength region, where only Water Crowfoot produces R^2^-values of over 0.5 for combinations of green wavelengths. Some of the lowest R^2^-values in this region (655 nm, 670 nm) are associated with chlorophyll absorption. All macrophyte species however still have some wavelength combinations in the VIS range that are significantly (99%) correlated with depth.

### 3.2. Depth—DN Relationships

**Figure 4:**
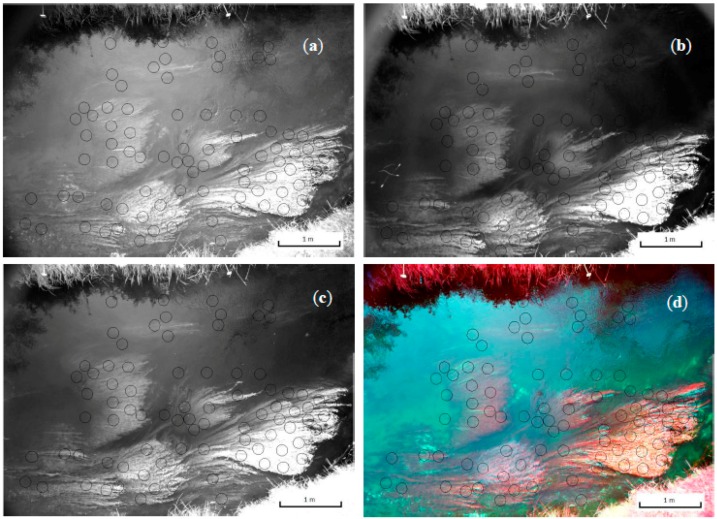
Image (composite) bands for site 1b: (**a**) NIR(BP1); (**b**) NIR(BP2); (**c**) NIR(R72); (**d**) False colour composite based on R, G, and NIR(R72). Black circles indicate depth/DN sample locations.

Figures 4a–d show a number of images obtained for site 1b. This includes the three NIR bands and a false colour composite composed of bands R, G, and NIR(R72). All images include the polygons from which DN samples were taken, which correspond with the depth measurement locations. Figure 9 contains RGB photos of all three sites. Cloudy weather at the time of sampling provided sub-optimal conditions for data collection. The reduced light availability resulted in reduced reflectance from the submerged vegetation surfaces and has affected the resulting water depth—DN relationships. Relative ambient light conditions are listed in Table 3.

**Table 3:** Time and light conditions during acquisition of image bands at each site.

Site No.	Approximate Sample Time	Filter	Range of Light Conditions (kLux)
1a	11:15–11:25	CC1, R72, BP1, BP2	43.3–47
1b	12:10–12:20	CC1, R72, BP1, BP2	75.9–79.3
2	13:40–13:50	CC1, R72, BP1, BP2	97.3–100.1
3	13:25–13:35	CC1, R72, BP1, BP2	108–123

**Figure 5:**
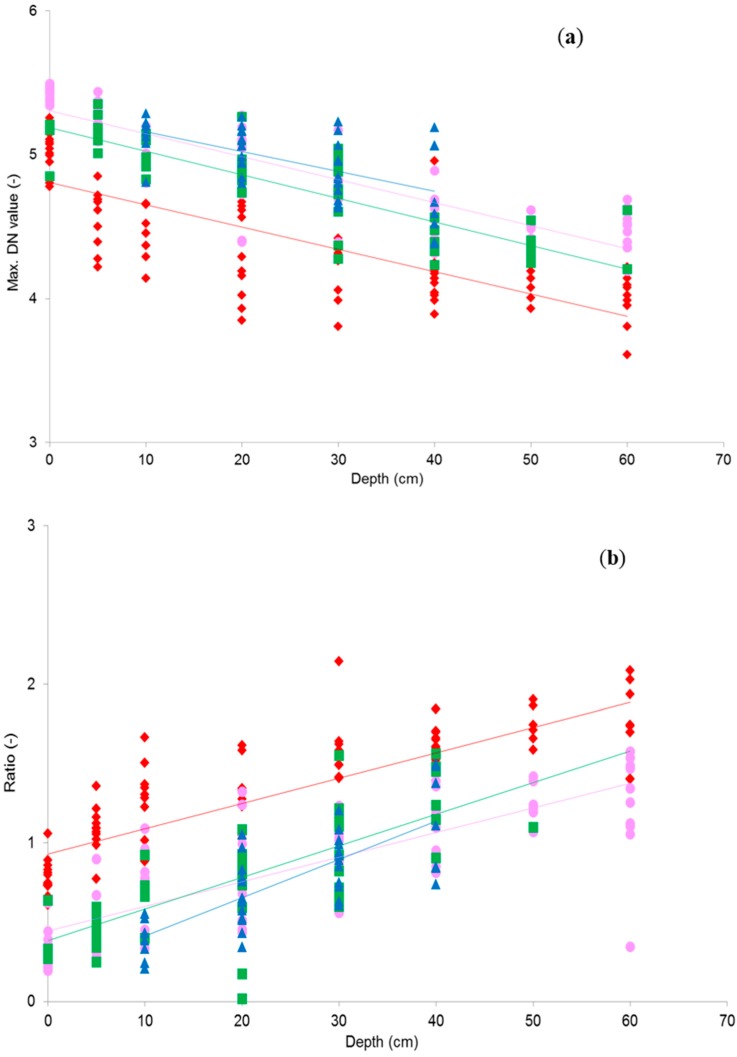
Natural logarithm of maximum DN values versus sample depth for samples from all sites for (**a**) NIR(R72); (**b**) Red/NIR(BP2) ratio. Sample numbers per site: site 1a (♦; n = 87), site 1b (●; n = 84), site 2 (■; n = 47) and site 3 (▲; n = 30). All shown model coefficients are significant at α = 0.05.

Figure 5a,b show a selection of the scatter diagrams that were created for water depth measurements versus the natural logarithm of the maximum pixel values of the spectral samples from the image bands and band ratios. Figures are included for NIR(R72) and the red/ NIR(BP2) band ratio. Each figure shows the results from all four measurement sites. For most bands and band ratios the maximum DN value statistic resulted in the most significant relationship with water depth, compared to other statistics. This was expected as reflectance from the vegetation surfaces are very heterogeneous due to gaps and shading. Pixel mean values will therefore be extremely variable and always under-represent top of canopy reflectance. Although maximum values may over-represent reflectance in case of exceptionally bright leaves or sunglint, these are thought to be minor errors.

**Figure 6:**
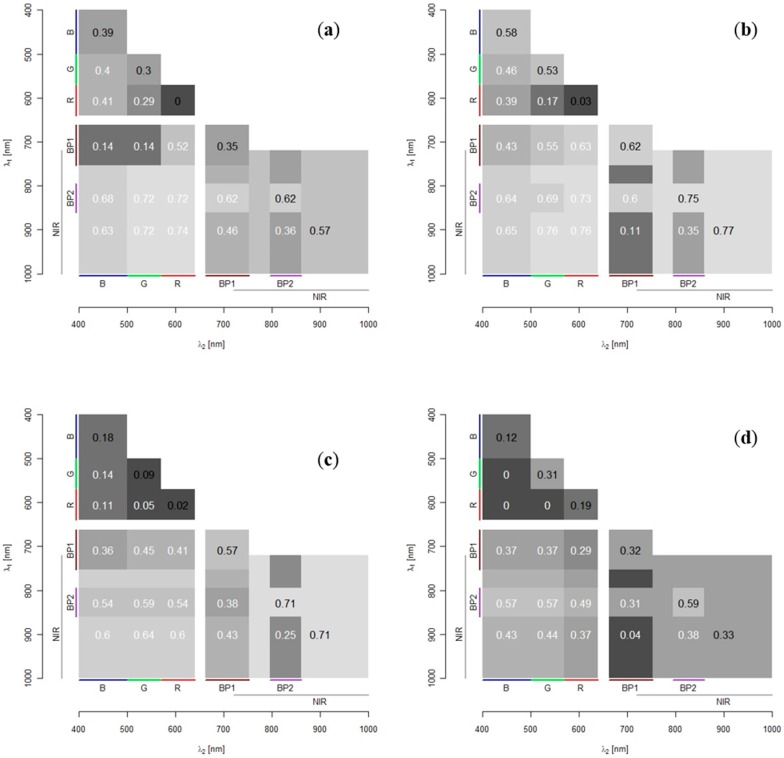
Optimal Band Ratio Analysis (OBRA) plots showing strength of image band ratio—depth associations for multi-spectral image composite bands and band ratios for (**a**) site 1a; (**b**) site 1b; (**c**) site 2; (**d**) site 3. Bottom half of each figure shows R² values of band ratios (white font). Diagonal values in black represent R² values for individual bands.

The distribution of R^2^-values amongst all wavelength bands and band ratios is illustrated in Figure 6 a–d, using the same approach as for OBRA in the first project phase. Two marked differences in this display are the inclusion of R^2^-values for the relationships of individual bands with depth on the central diagonal line of the plot and the presence of overlapping bandwidths, in this case NIR(72) and NIR(BP2). The figure shows clear differences in the strength of the relationships with depth amongst the individual wavelength bands and band ratios. Compared to the NIR bands, the VIS bands perform clearly less well. Although the blue band shows strongest associations when used on its own, it performs less well when used in a band ratio with most of the other bands. The red and green bands on the other hand perform poorly individually, while they show some of the strongest associations when combined with NIR bands. The best performing individual infrared bands are NIR(R72), covering the whole NIR region, and NIR(BP2), which is centred around 828 nm. The Red/NIR(R72) band ratio seems to slightly outperform the Red/NIR(BP2) combination, though the differences are very small and vary across the measurement sites.

The band (ratio)—depth relationships found for the different sites tend to have similar slopes. However, in particular the intercept of site 1a for most band (ratios) is considerably lower than those of the other three sites. This difference is expected to be due to the illumination conditions during the sampling of this site. Samples for site 1a were taken at the same location as site 1b, so between these two sites other factors influencing the results should be limited. Further improvement of light conditions during sampling at site 1b, 2, and 3, was expected to result in slightly higher reflectance at site 2 compared to site 1b, but this was not the case. Additional influences, perhaps the different, brighter, and shallower vegetation on site 1b, may have been the reason for this.

### 3.3. Depth Modelling

Observations from site 1b were assumed to provide the most reliable reflectance-depth model. Not only does the site quite consistently show the highest R^2^-values, it also contains the rather dense Water Starwort, which is likely to provide more consistent reflectance values than the loose leaves of Broad-Leaved Pondweed at sites 2 and 3. Although the associations based on band ratios were generally weaker than for some based on individual bands, the best of each was used for further depth mapping. A band ratio based relationship was included as it was expected to provide a more reliable basis for a bathymetric model compared to individual bands. The regression equations for band NIR(R72) and band ratio Red/NIR(BP2) from site 1b were selected for further depth modelling. Although band ratio Red/NIR(R72) for most sites shows a slightly stronger association with depth, the differences with the Red/NIR(BP2) band ratio are minimal. The main reason to choose Red/ NIR(BP2) band ratio is that the wavelength range covered by this band combination (red: 570–645 nm; NIR(BP2): 795–860 nm) overlaps closely with the findings from OBRA in phase one of the project. This showed that band combinations including both a NIR band between 825 and 925 nm and a band in VIS had the strongest association with water depth. The selected relationships were significant at 99%.

The regression equations used for mapping of depth (d_e_) were as follows for the ratio based equation, which used the Red/NIR(BP2) combination:

d_e_ = (ln(DN_Red_/DN_NIR(BP2)_) − 0.4439)/0.0155 (R² = 0.73) (4)

and the equation for the single band, using NIR(R72):

d_e_ = (ln(DN_R72_) − 5.3061)/ (−0.016) (R² = 0.77) (5)

### 3.4. Model Validation

**Figure 7:**
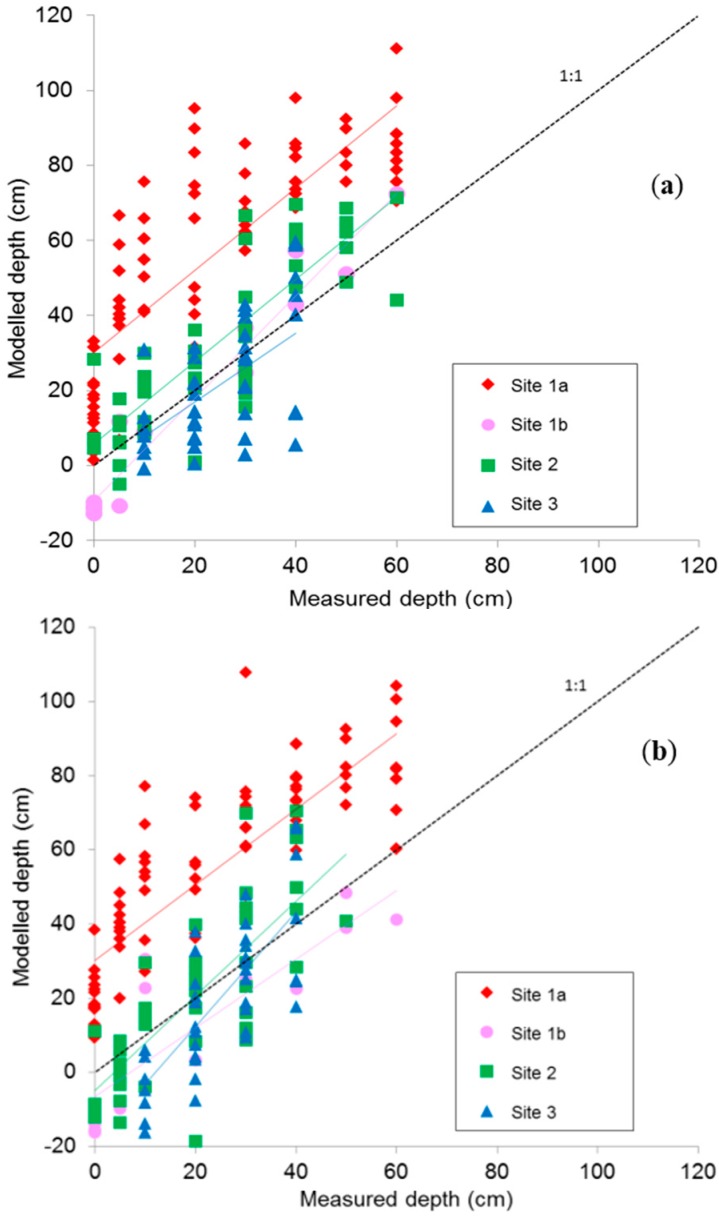
Depth estimated with (**a**) NIR(R72) model and (**b**) Red/BP2 ratio model versus measured depth for samples from site 1a (♦; n = 87), site 1b (●; n = 84), site 2 (■; n = 47) and site 3 (▲; n = 30). Dashed line indicates 1:1 line. All shown model coefficients are significant at α = 0.05.

The scatter diagrams of Figure 7 compare the depth values estimated with both models for the 15% data excluded from the model development and the values measured in the field. The validation data show high R^2^-values for both models (0.74 for Red/NIR(BP2) and 0.95 for NIR(R72)), which suggest a good agreement between model predictions and reality, but they do not exactly follow the 1:1 line that would confirm accurate prediction. The Red/NIR(BP2) band ratio model seems to slightly under predict depth. In the case of NIR(R72) model, the lower depth values are under predicted and the higher values over predicted. In both cases the results may be influenced by a large number of observations at the water surface, some of which have relatively higher spectral values, because the vegetation is not actually covered by a water layer. Under uncovered conditions the relationship will not be valid.

Figure 7a,b also contain scatter diagrams of measured and predicted depth values that result from applying the model to the sample points of site 1a, 2, and 3. They confirm the pattern that would be expected from the original data. Both models clearly overestimate the depth values for site 1a, as they associate the low DN values in this image with deeper water. The values for site 2 are also slightly overestimated, although this cannot be directly explained by the light conditions. Depth values for site 3 hover around the 1:1 line and the site 1b results, which suggests that the predictions are quite good. The slope of the regression is however different, which indicates that there are other complicating factors.

**Figure 8:**
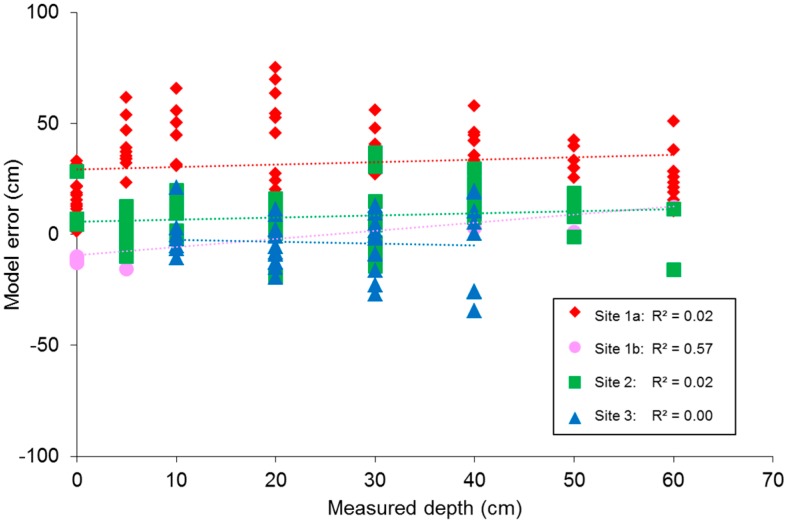
Scatter diagram of measured depth versus NIR(R72) model error for samples from site 1a (♦; n = 14,), site 1b (●; n = 84, validation data only), site 2 (■; n = 47) and site 3 (▲; n = 30).

The mean of the absolute depth error $\left(\Delta ̅\bar{\text{y}}\right)$ is 32.4 cm for estimates of site 1a depth with the NIR(R72) model. $\Delta ̅\bar{\text{y}}$ for the other three sites range between 9.0 and 11.5 cm. The $\Delta ̅\bar{\text{y}}$ for site 1a depth estimates with the Red/NIR(BP2) model is 30.6 cm while the values range between 12.1 and 13.5 cm for the other three sites. Figure 8 shows the measured depth versus model error of the NIR(R72) model for the data from each site. For site 1b only validation data was used. For this site the diagram suggests there is a significant association between depth and model error (α = 0.05). However, no significant relationships were found when the model was applied to any of the four full datasets, so we expect that result is not representative and assume there is no depth bias in the model results.

### 3.5. Depth Mapping

**Figure 9:**
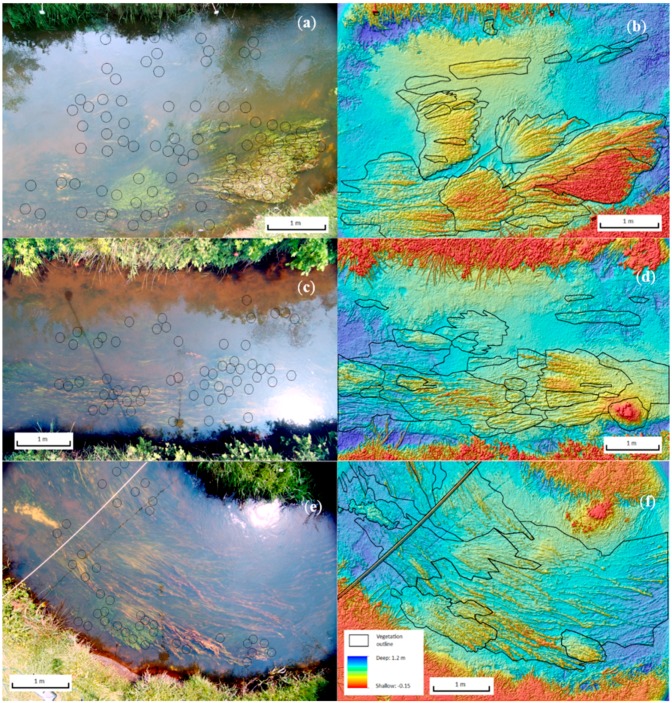
RGB composite of site 1b (**a**); DEM of site 1b based on NIR(R72) ratio-model (**b**); RGB composite and DEM based on NIR(R72)-model for site 2 (**c**,**d**); RGB composite and DEM based on NIR(R72)-model for site 3 (**e**,**f**). All DEMs have been smoothed with a 5 × 5 filter. Hillshade is added to enhance the visibility of the relief. Black lines show manually digitized outlines of vegetation patches. Legend is included in Figure 9f.

DEMs were calculated for each site using the two best models derived from the data of site 1b (Equations (4) and (5)). The resulting DEMs clearly show the superior performance of the NIR(R72) model (Figure 9b). The ratio based DEM is strongly affected by the fact that its layers were not taken simultaneously. Movement in the vegetation between layer acquisition causes clear ‘noise’ in the data as it causes short distance contrast in ratio values. Figure 9 d,f show the DEMs for the other two sites, again based on the NIR(R72) model. The topographic data created for the three sites appear highly realistic. The modelled depths range from around 5 to 105 cm, so the error is approximately 10% of this depth range.

### 3.6. Preprocessing Evaluation

While it was possible to manually exclude cells affected by sunglint from the analysis, removing the radiometric effects of flaring was more difficult. Removal based on an image-derived mask was attempted for a strongly affected NIR(BP2) image of site 1b. Minimum DN values were extracted from the affected image as described in Section 2.4.1. After interpolation the values showed a sinusoidal pattern, as well as a trend of decreasing radiometric values away from the centre of the image. The latter observation suggests that there may also be some vignetting occurring.

**Figure 10:**
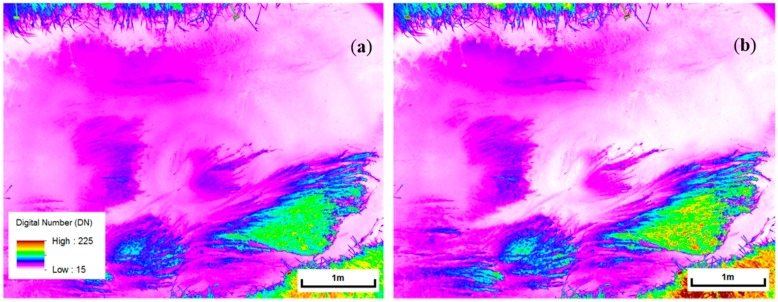
Comparison of NIR(BP2) image for site 1b before (**a**) and after (**b**) radiometric correction of flare effect (‘temperature’ style colour ramp used to enhance DN variation).

The pattern was extrapolated across the image area to create a mask, which was subtracted from the original image. This clearly reduced the flaring/vignetting effect, as can be seen in the image close-ups in Figure 10. Alternative depth models were calculated for the corrected data. The R^2^ of the model for site 1b based on NIR(BP2) data (n = 84) actually reduced from 0.75 before to 0.72 after radiometric correction of the flare effect. When the correction method was applied to the NIR(BP2) image of site 2, the result was even less successful, as the image shows a clear shadow area on the water, which results in extra low minimum DN values for the circles crossing this area. The correction is clearly affected by this. Since the correction did not clearly improve the model results, no further attempts have been made to make corrections for the remaining images. All subsequent analysis results are based on data corrected for sunglint only.

## 4. Discussion

### 4.1. OBRA Band Selection

The OBRA analysis, based on the field spectroscopy data, showed that good estimates of SAV submergence depth should be possible based on a combination of VIS and NIR bands. Slight variations exist in best performing band combinations between individual SAV species, with Water Crowfoot showing the strongest overall water depth–band ratio association. The combined assessment of all species, however, results in associations of similar strength as most individual species for an equally wide range of wavelength combinations. This indicates that the effect of spectral variation in vegetation is subsidiary to spectral variation due to depth changes and a single model should provide sufficiently accurate results for many applications that require information on spatial distribution of SAV.

The OBRA plots show some similarities with those of [15], for example the low R^2^ values at the chlorophyll absorption feature around 675 nm. However, the highest associations are found for rather different wavelength combinations. Legleiter *et al*. [15] found that the log transformed band ratio *X* of reflectances at 586 and 614 nm was strongly related to depth (R^2^ = 0.945) independent of suspended sediment concentrations, *R_b_(λ)*, and water surface state. For our vegetation examples, wavelengths in this region have consistently very low R^2^ values (significance <95%). Instead a slightly unexpected combination of one band in the NIR region between 825 and 925 nm and one band in the VIS region, was found to have the highest correlation values. In [15] the NIR bands of simulated reflectance spectra of substrate at different submergence depths were not selected as suitable for depth retrieval. They assumed this to be due to saturation of the radiometric signal at greater depths. For spectra measured in the field, which covered a smaller depth range, NIR bands were identified as most useful for depth retrieval (although wavelength ranges measured did not go beyond 900 nm). Though the saturation effect should be even greater for the longer wavelengths selected in this study, it apparently does not affect the vegetation signatures within the limited depth range of the chalk streams observed in this study.

### 4.2. Depth Mapping with Multispectral Data

Testing the best band ratios found for depth mapping through OBRA would require hyperspectral image data at a spatial resolution that allows delineation of SAV patches. This kind of data is currently not commonly available. Instead six-band multi-spectral image composites were created, using a NIR sensitive DSLR camera. Based on a field dataset of SAV submergence depths, regression models were developed that enabled mapping of submergence depth from image DN values. The results from this analysis confirmed the previous OBRA findings, with similar combinations of VIS and NIR providing strongest associations. However, NIR single band associations with depth resulted in even better fits. Overall, it has become clear that it is possible to map submerged vegetation bathymetry, using optical image data obtained with a consumer grade camera. This study also showed that it does not necessarily require combinations of very narrow hyperspectral bands to do this, but that good results were possible with broadband data of high spatial resolution. Under sub-optimal data collection conditions (as discussed below) the mean of the absolute depth error was within 10% of the observed depth range, which is thought to be satisfactory. However, we do need to stress that there was considerable variability in the error observed. So an individual depth estimates obtained for a specific point can still be quite far off the true submergence depth. A considerable benefit of the novel proposed approach is the very low cost of data acquisition.

In this study, the decision was made to use the model derived from the image taken under the ‘best’ conditions to estimate depth for all sites. In the case where water column, water surface, and weather conditions were constant for all sites this would mean that only the spectral variation in vegetation surface reflectance would affect model performance. Unfortunately the rather variable weather/illumination conditions do not allow to attribute the discrepancies found between measured and predicted values to this factor only. However, the use of a band ratio in one of the two models was expected to eliminate variation in the magnitude of model error between the sites due to differences in surface cover types [19]. Since the ratio based model showed very similar variation between the sites compared to the single band model, the effect of vegetation variation is expected to be minimal. The ‘best’ estimate models may therefore be suitable for wider application in vegetated streams, though further investigation of the impact of other factors on the model is needed.

### 4.3. Limitations of Data Collection and Analysis

A number of sensor characteristics of the consumer grade digital camera and filters used for image data acquisition, as well as the procedures chosen for image analysis may have affected the outcomes of this study. They are discussed in the following sections.

#### 4.3.1. Sensor Platform and Image Registration

The presented approach uses a pole fixed with guy ropes as camera platform. This set-up can be installed in less than 15 min, but is not suitable for covering larger river sections. More portable pole platforms are possible [25], but ideally full river reaches will be photographed from a UAV. Further research is needed to test how well the models perform on the lower resolution imagery obtained from such more elevated kind of platform. All suggested platforms have in common that they require co-registration of image bands taken at different times. This is a major weakness, as the process is time consuming and a perfect match is impossible in the continuously moving submerged environment. Some registration improvement may be achieved by increasing the number of ground control points, but is likely to be insignificant compared to the error due to plant moment. The resulting mismatches between bands create an unwanted texture in the DEM as demonstrated in the ‘Depth mapping’ section. The problem can be resolved by using multiple synchronized cameras [26]. In this project it was not an option as we needed to test multiple filters and handling more than one FujiFilm DSLR cameras at a time would have been challenging. However, when optimal filter combinations are known, a setup with two or three NIR converted compact cameras may suffice. Overall, we do not think that ‘misregistration’ will have had an influence on the final conclusions drawn from this research, as the 2 cm error becomes insignificant within the 18.5 cm diameter sample polygons.

#### 4.3.2. Spectral Resolution

Furthermore the spectral resolution of the data was low, so the exact bands determined with the OBRA method could not be used. The fact that the closest possible combination provided the best results is encouraging. Interference filters do exist that allow image acquisition in spectral bands much narrower than the broad bands used here. Filters for wavelengths that corresponded better with the results obtained in the OBRA study may result in models with better R^2^ values. However, the best band combinations do vary for different types of vegetation, so it is possible that the broad band filters allow for better overall results. Narrow bandpass filters require more sophisticated optical systems to ensure a consistent spectral response [27]. The image quality was already quite low for the NIR(BP2) bandpass filter.

#### 4.3.3. Image Compression

Shooting 12/14 bit RAW rather than TIFF images may improve analysis results and is recommended for scientific applications [22]. However, practical application is compromised while some studies have shown that compression does not necessarily affect results when doing radiometric assessment using consumer grade cameras [23]. Researchers regularly use even poorer quality (JPEG) data with good results as the larger RAW file sizes may not be suitable for continuous shooting from UAV platforms (e.g., [28]).

#### 4.3.4. Flaring or Hot-Spotting and Other Filter Related Issues

In particular the images of band NIR(BP2) were affected by a phenomenon called ‘flaring’, which is caused by internal reflection of light between the camera lens and some of the applied filters [29], resulting in circular shaped variation in pixel brightness (see Figure 4b). An attempt to remove the effect from one of the NIR(BP2) images did not seem to improve the model fit. It reduced the depth dependency of the DN value. This could be partly due to the fact that water depth decreases in most directions away from the centre of the photo, while the mask DN values also decrease away from highest values in the centre. Subtraction should therefore lead to a reduction in the depth-DN relationship. So, although the radiometric correction removed a trend and sinuosity in the data it did not clearly improve the results. Complex shading and reflection patterns also made the pre-processing approach less suitable for some other images. It was therefore not used to obtain final map products. Improvements can be made to the model results by finding a camera/lens/filter combination which does not produce similar radiometric anomalies.

#### 4.3.5. Diffuse Light, Surface Reflection and Shading

Due to the mostly cloudy weather during image data collection, diffuse light reflection at the water surface was clearly visible in the VIS wavelength band images. This may have affected the model performance in these wavelengths; however the results from the OBRA part of the study already indicated the limited use of VIS wavelength for SAV depth mapping. NIR wavelength bands were visibly less affected by reflection, though the lower light intensity still lowered the model intercepts.

**Figure 11:**
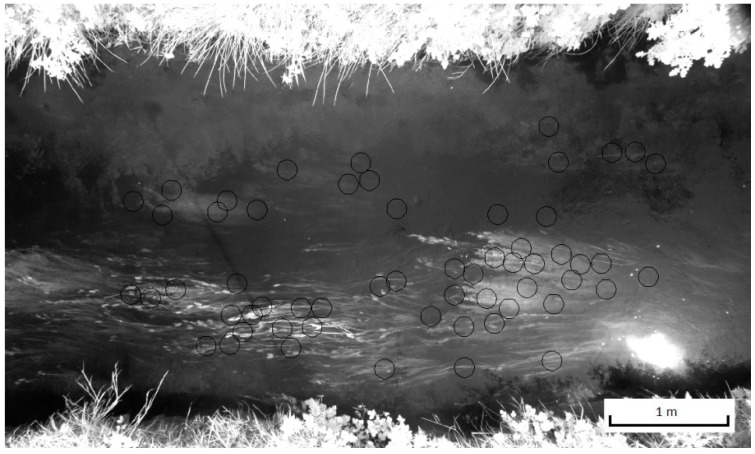
NIR reflection at water surface (light haze in top half of the reach); Band NIR(R72), site 2.

A more complex problem is caused by a combination of shading and water surface reflection. Reflection causes ‘images’ from the surrounding landscape elements on the water surface. In particular the NIR bands clearly show bright pixels that represent reflection of vegetation on the river banks. This effect is best visible in the NIR(R72) band of site 2 (Figure 11). The relatively high NIR(R72) values in the top half of the photo, where reflection of vegetation at the water surface was strongest, resulted in a clear underestimation of the water depth (and thus a higher DEM surface elevation) as can be seen in Figure 10d. Shading of the water surface by the river bank, causes an opposite problem, which can be seen most clearly in the DEM for site 1b based on the NIR(R72) model (see Figure 9b). In the DEM for this site a strip of water next to the top bank shows clearly overestimated water depths (approximately 0.4 m). Errors of similar magnitude due to shading effect on water depth estimates were found in [16] for the Laramie River in Colorado, US.

Since the spectral signatures of the water surfaces with vegetation reflection and shading will form complex mixtures with to those of the submerged vegetation and other substrate [30] it will be difficult to mask out their effect. However, since the effect occurs mostly along the edges of the water and most depth measurements were taken more towards the middle of the stream cross sections, the effects on model development are thought to be small. The effect on DEM mapping are however important. While in-air and in-water adjacency effects have been observed and investigated for some time in fluvial [15,16,30], lake, and coastal [31] environments, no solution to either of these problems is however currently known. Clearly further work is needed to find suitable correction methods.

#### 4.3.6. Bidirectional Reflectance Distribution Function (BRDF)

A commonly occurring problem with remote sensing of vegetation is the bidirectional reflectance distribution function (BRDF) effect. As vegetation is illuminated from different directions its reflectance will be different at different points in the image. However, by using the highest values from our spectral samples rather than average values this problem will to some extent be limited. In addition to this the camera was in vertical position for all photos. Various studies have shown how BRDF only becomes significant under sufficiently large viewing angles (e.g., [32]) and exactly how the effect works in submerged environments is an area of ongoing research.

### 4.4. Application Potential

This study focused on the potential to map submergence depth of SAV using a consumer grade camera as sensor. The results show that in sufficiently shallow and clear streams there is the potential to create a combined model for both vegetated and non-vegetated surfaces to map the full river bed bathymetry including the vegetation. It will depend on the application of the model whether a 10% error is acceptable. A clear limitation of the method presented here is that it can only provide a ‘2.5D’ representation of the vegetation structure. The spectral data cannot give information on what happens below the plant surface. Additional thought is required on how to transfer the information satisfactorily into 3D flow models.

Another limitation is the depth at which this method will work. Lejot *et al*. [13] found that the method could measure bottom depth up to 5 m using VIS wavelengths. For NIR based estimates this will be less due to the stronger attenuation of these wavelengths. However, as SAV requires light to thrive, most activity/biomass of SAV will be present in shallow and relatively clear water only.

The OBRA results were obtained from different species than present in the depth mapping exercise. Similar wavelength regions were found to be useful and several species were included in the depth mapping exercise. We therefore assume that the model is applicable to a wider range of vegetation species, but further testing is required here.

Most studies of submerged vegetation in marine and other aquatic environments do not include the wavelength bands of the NIR. This study showed that the NIR wavelengths and in particularly those beyond 800 nm contain some important information, which seems to be different from land-based vegetation where normally the red edge is used to gain information about vegetation type and health. In [33] it was already pointed out that the NIR bands are also useful for SAV species detection. Although these findings may not apply to marine environments which are generally much deeper, it seems important to incorporate reflectance estimates beyond 800 nm when studying submerged environments with abundant SAV.

## 5. Conclusion

As consumer grade cameras are still the most commonly used sensors on low-altitude remote sensing platforms such as UAVs more research into their use to map, for example, the spatial distribution of fluvial properties is warranted. In particular, in small river systems image data collected with such cameras can be of great use. The results of this study show specifically how mapping the extent and submergence depth of submerged aquatic vegetation (SAV) shallow clear water streams is feasible.

The study firstly identified which wavelength bands are most useful for spectrally based bathymetric mapping of SAV. A remarkable finding was that in particular wavelengths between 825 and 925 nm are important, if not necessary for this purpose. This was not expected as this wavelength region is generally thought of little use, because of the very strong absorption of water in this wavelength region and it is very different from the most suitable bands found for non-vegetated river bottoms [15].

In addition to this, the study showed that reasonably accurate estimates can be made of SAV submergence depth distribution (with an average error of 10% of flow depth range), using multi-spectral image data that was easily and cheaply obtained with a near infrared (NIR) sensitive DSLR camera and a set of (bandpass) filters. The wavelength bands that were found to be most suitable for this purpose, were obtained with a VIS blocking filter (<720 nm) and a bandpass filter with maximum transmission between 795 nm to 860 nm. These findings correspond with the OBRA results, confirming the benefit of spectral information from these wavelength regions for SAV depth mapping

Although spectrally based bathymetric mapping techniques have previously been successfully applied in non-vegetated streams, the findings presented in this paper make an important contribution to the development of these techniques by demonstrating how results are different in the presence of submerged aquatic vegetation (SAV). To obtain a full picture of submerged river environments it is important to make a link between the studies that looked at non-vegetated river reaches [16] and develop methods that allow simultaneous mapping of vegetated and non-vegetated surfaces, by combining the findings from both environments. This potentially requires procedures that can accurately mask-out vegetated from non-vegetated areas, so that different band ratios can be applied to each in order to obtain the most accurate river bed maps.

A much greater challenge will be obtaining a true 3D representation of the vegetation. The spectrally based method only provides a so called 2.5D version of reality. It does not allow us to see within or underneath the aquatic plant canopy. SAV species can have very diverse morphology and therefore respond differently to the surrounding flow/hydrology. Further research is required to find approaches that can more accurately represent this information.
